# *Paracentrotus lividus* sea urchin gonadal extract mitigates neurotoxicity and inflammatory signaling in a rat model of Parkinson’s disease

**DOI:** 10.1371/journal.pone.0315858

**Published:** 2024-12-18

**Authors:** Nehal Shawky Nagy, Mohamed Helal, Eman Sheta Alsawy, Mohamad Moustafa Ali, Soheir Salem Al-Sherif, Amina Essawy Essawy

**Affiliations:** 1 Faculty of Science, Department of Zoology, Alexandria University, Alexandria, Egypt; 2 National Institute of Oceanography and Fisheries (NIOF), Cairo, Egypt; 3 Department of Biology, University of Southern Denmark, Odense, Denmark; 4 Faculty of Medicine, Department of Pathology, Alexandria University, Alexandria, Egypt; 5 Department of Medical Biochemistry and Microbiology, Science for Life Laboratory, Uppsala University, Uppsala, Sweden; IMS-BHU: Banaras Hindu University Institute of Medical Sciences, INDIA

## Abstract

The present study investigates the neuroprotective effects of the sea urchin *Paracentrotus lividus* gonadal extract on rotenone-induced neurotoxicity in a Parkinson’s disease (PD) rat model. Parkinson’s disease, characterized by the progressive loss of dopaminergic neurons in the substantia nigra (SN), is exacerbated by oxidative stress and neuroinflammation. The study involved fifty Wistar rats divided into five groups: control, dimethyl sulfoxide (DMSO) control, *Paracentrotus lividus* gonadal extract-treated, rotenone-treated, and combined rotenone with *Paracentrotus lividus* gonadal extract-treated. Behavioral assessments included the rotarod and open field tests, while biochemical analyses measured oxidative stress markers (malondialdehyde (MDA), nitric oxide (NO), glutathione (GSH)), antioxidants (superoxide dismutase (SOD), catalase (CAT)), pro-inflammatory cytokines (interleukin-1 beta (IL-1β), interleukin-6 (IL-6), tumor necrosis factor-alpha (TNF-α)), and neurotransmitters (dopamine (DA), levodopa (L-Dopa)). Histological and immunohistochemical analyses evaluated the neuronal integrity and tyrosine hydroxylase (TH) and alpha-synuclein expression. The results showed that *Paracentrotus lividus* gonadal extract significantly mitigated rotenone-induced motor deficits and improved locomotor activity. Biochemically, the extract reduced oxidative stress and inflammation markers while enhancing antioxidant levels. Histologically, it restored neuronal integrity and reduced alpha-synuclein accumulation. Molecularly, it increased tyrosine hydroxylase and dopa decarboxylase gene expression, essential for dopamine synthesis. These findings suggest that *Paracentrotus lividus* gonadal extract exerts neuroprotective effects by modulating oxidative stress, neuroinflammation, and dopaminergic neuron integrity, highlighting its potential as a therapeutic agent for Parkinson’s disease.

## Introduction

Neurodegeneration is characterized by the progressive loss and dysfunction of neurons and axons of the central nervous system due to oxidative stress, contributing notably to dopamine cell degeneration, as evident in Parkinson’s disease (PD) [1–3]. PD is a prevalent movement disorder frequently encountered in neurological practice. It is the second-most common neurodegenerative disorder that manifests as a familial or sporadic form. The etiology of the disease remains largely unknown; however, it is believed to result from a combination of genetic and environmental factors [4]. The most common neuropathological hallmarks are neuronal loss in the substantia nigra, leading to striatal dopamine deficiency, and intracellular inclusions containing aggregates of α-synuclein [5]. These α-synuclein aggregates, found in Lewy bodies or Lewy neurites, disrupt subcellular transport mechanisms that are dependent on microtubules, leading to synaptic dysfunction and neuronal homeostasis imbalance [6,7]. PD presents numerous clinical challenges, including early diagnosis and symptom management in advanced stages. Currently, there are no available treatments that can ameliorate the neurodegenerative process, additionally, the long-term administration of chemical drugs is often accompanied by severe side effects, and none of these drugs can halt or reverse PD progression [8,9].

In contrast to synthetic drugs, bioactive compounds derived from natural sources are preferred due to their natural origin and decreased side effects. Natural products derived from medicinal plants, fruits, and vegetables have been shown to effectively alleviate PD symptoms in animal models [10]. These natural products exhibit neuroprotective properties due to their well-known anti-inflammatory and antioxidant activities. They also inhibit iron accumulation, protein misfolding, and proteasomal degradation [11,12]. Marine-derived natural compounds are promising for their diverse pharmacological effects, making them valuable in drug discovery and development [13,14]. Marine invertebrates, in particular, represent a rich source of bioactive compounds with therapeutic potential, targeting specific molecular pathways involved in diseases such as neuroinflammation and neurodegenerative disorders [15]. Among different invertebrates, sea urchins, members of the class Echinoidea, are benthic echinoderms abundant in antioxidants, anti-tumor, and antimicrobial agents [16–18]. The edible portion of sea urchins consists of their yellow to orange, half-moon-shaped gonads, prized for their distinct flavor, richness in bioactive compounds, and medicinal properties [19]. Numerous studies have investigated the neuroprotective effects of sea urchin extracts on various neurodegenerative diseases. For instance, Echinochrome A, isolated from the sea urchin *Scaphechinus mirabilis*, has demonstrated therapeutic potential in reducing acetylcholine-related diseases such as Alzheimer’s disease (AD) [20]. Gangliosides from the sea urchin *Strongylocentrotus nudus* promote AD resistance in vivo and in vitro by reducing neurite loss and preventing cellular apoptosis [21] and extracts from the sea urchin *Diadema savignyi* exhibit strong neuroprotective activity against cisplatin-induced neurotoxicity in rats [22].

*Paracentrotus lividus* (Lamarck, 1816), the most edible among sea urchin species, inhabits the Atlantic and the southeastern Mediterranean coast of Alexandria, Egypt [23]. Previous studies have demonstrated the anti-inflammatory, gastroprotective, analgesic, antimicrobial, and anti-obesity activities of various *P*. *lividus* extracts [24–26]. To the best of our knowledge, the efficacy of sea urchin gonad extract against a PD animal model has not yet been explored. Given its pharmacological properties, our study aimed to investigate the neuroprotective effects of *P*. *lividus* gonadal extract against rotenone-induced neurotoxicity caused by mitochondrial respiratory chain complex I dysfunction [27] in rats. Moreover, we aimed to determine the potential of this gonadal extract as a neuroprotective agent to mitigate Parkinson’s disease symptoms by assessing its effects on behavioral, neurochemical, molecular, and histopathological alterations induced by rotenone.

## Materials and methods

### Chemicals and reagents

High analytical grade chemicals and reagents were used in this study. Rotenone (Rot) (C23H22O6 ≥ 98% purity) was obtained from Sigma-Aldrich (St Louis, MO, USA). Reagents for malondialdehyde (MDA, Cat. No. MD2529), nitric oxide (NO, Cat No. NO2533), glutathione reduced (GSH, Cat. No. GR2511), superoxide dismutase (SOD, Cat. No. SD2521) and catalase (CAT, Cat. No. CA2517) were obtained from Biodiagnostic (Cairo, Egypt). Rat interleukin 1 Beta (IL-1β) ELISA Kit (Cat. No. E0119Ra), Rat interleukin 6 (IL-6) ELISA Kit (Cat. No. E0135Ra) and Rat Tumor Necrosis Factor Αlpha (TNF-α) ELISA Kit (Cat. No. E0764Ra) were obtained from Shanghai Korain Biotech BT-lab (Shanghai, China). Dopamine (DA) ELISA Kit (Cat. No. E-EL-0046) was obtained from Elabscience (Texas, USA). Rat l-dihydroxyphenyalanine (L-DOPA) ELISA Kit (Cat. No. MBS9357024) was obtained from MyBioSource (San Diego, USA). For gene expression, TriRNA Pure Kit with DNase (Cat. No. TRP050/D050) obtained from Geneaid (Taipei, Taiwan), TOPscript™ cDNA Synthesis Kit (Cat. No. EZ005S) obtained from Enzynomics (Daejeon, Korea) and Maxima SYBR Green qPCR Master Mix (2×) (Lot. No 01204048) obtained from Thermo Fisher Scientific™ (USA).

### *P*. *lividus* gonadal extract preparation

Mature adults of *P*. *lividus* (Lamarck, 1816) were collected by marine divers from the Mediterranean coast of Alexandria (Abou Quir), Egypt. Sea urchins were dissected, gonads were collected, and total gonadal extract was prepared according to the method previously described [18]. Briefly, gonads were weighed and washed in seawater to remove debris. For each three to four grams of weighted gonads, ten ml of HPLC grade acetone was added and gentle homogenization was carried out on ice. The homogenate was collected and centrifuged for three minutes at 1500 rpm under cooling conditions. Then, an equal volume of methyl tertiary-butyl ether (MTBE) and five ml of distilled water was added to the homogenate and vigorously shaken. The organic layer (which contains the extract) was collected, filtered, and dried under nitrogen gas evaporation. The extract was stored at -20°C until further application.

### Animal subjects and experimental design

Wistar male rats weighing 180 ± 10 gm were obtained from the animal care unit, faculty of medicine (Alexandria University, Egypt). Animals were randomly divided into five groups and kept under a 12 h light-dark cycle at a temperature of 23 ± 2°C, and humidity (50%–60%) and with free access to food and water. Animals were allowed to adjust to housing for at least one week before the experiment was initiated. All experimental procedures and animal handling were aligned with the guidelines approved by Alexandria University Institutional Animal Care and Use Committee (ALEXU-IACUC), a member of the International Council for Laboratory Animal Science (ICLAS) (Approval number: AU 04 21 09 23 2 01).

Fifty rats were randomly divided into five groups with ten animals in each group and treated as follows: the control group was orally administered corn oil (0.5ml) as a vehicle control, the DMSO control group (0.2 ml, subcutaneous injection), *P*. *lividus* gonadal extract treated group (dissolved in corn oil as 30 mg/kg, oral administration), Rotenone treated group (dissolved in DMSO as 2 mg/kg, subcutaneous injection) and Rot + *P*. *lividus* gonadal extract treatment group (30mg/kg gonadal extracted supplemented by 2mg/kg rotenone after one hour). The Dose of Rot were determined according to previous studies [28]. Depending on the obtained results from our previous study [18], Ic50 for the *P*. *lividus* gonadal extract was calculated, in the recent study we used the Ic50 to calculate LD50 using the regression formula given by the interagency Coordinating Committee on the Validation of Alternative Methods (ICCVAM): log LD50 (mg/kg) = 0.372 log IC50 (μg/mL) + 2.024 [29,30]. the lowest dose of *P*. *lividus* gonadal extract was used in the recent study. The rats were treated for six weeks daily.

### Behavioral studies

#### Rotarod test

The motor coordination of rats was assessed by using the accelerating rotarod [31,32]. Rats were trained for three days before testing their ability to walk on an accelerating rotarod cylinder using accelerating speed levels (5,10,15, and 20 rpm) Following the training, rats were put on the rotarod cylinder for testing at 20 rpm speed for three minutes. Latency to fall was recorded for each rat three times and the mean value was calculated.

#### Open field test

Spontaneous locomotion of rats was assessed by performing the open-field test [33]. Rats were placed in an open field square arena and the testing duration was five minutes for each rat. Ambulance frequency (the number of squares the animals crossed during the test) and Rearing frequency (the number of times the animals stood on their hind feet) were recorded. To remove possible odors left by other animals, the arena was washed with a 5% water-alcohol solution before and between the open field tests.

### Tissue sampling

Once the two behavior assessments were accomplished, the animals from each group were anesthetized by intraperitoneal injection of a mixture of xylazine and ketamine (ketamine; 100mg/kg + xylazine; 5mg/kg) and sacrificed through cervical dislocation. The brains of five rats from each group were quickly removed and dissected to isolate the nigrostriatal tissue for biochemical analysis. At the same time, the brains of the other five rats were extracted and cut into two halves. The nigrostriatal tissue of the right halves was fixed in 10% formalin for histological and immunohistological studies, while those of the left halves were stored at -80°C for gene expression analysis.

### Biochemical analysis

Striatum and substantia nigra from five rats were isolated, perfused with phosphate-buffered saline PBS (pH 7.4), and homogenized in cold sucrose buffer (0.25 M) using tissue homogenizer at 4000 rpm for 15 minutes at 4°C. The supernatant was collected and used for the assessment of oxidative stress markers (MDA, NO & GSH), antioxidants (SOD& CAT), pro-inflammatory cytokines (IL-1β, IL-6) and TNF-α) and neurotransmitters (DA and L-DOPA). Livre and kidney functions were assessed to study extract toxicity in the control, DMSO, and extract-treated group (S1 Table).

### Assessment of oxidative stress, inflammatory biomarkers, and neurotransmitters

To evaluate the lipid peroxidation in the brain homogenates, MDA was estimated, as described previously [34], nitrosative stress was measured according to the Griess method [35] in which endogenous nitrite concentration was measured as an indicator of nitric oxide production, while GSH was assessed by the colorimetric determination method according to the manufacturer’s instructions. The activities of SOD and CAT were estimated following the procedures of [36,37], respectively. The inflammatory cytokines IL-1β, IL-6, and TNF-α were measured using rat ELISA kits and the analyses were done according to the manufacturer’s instructions. Dopamine (DA) and L-DOPA levels were estimated using the relevant ELISA kit instructed by the manufacturer’s protocol and measured as ng/g of brain tissue.

### Histopathological and immunostaining analysis

Striatum and substantia nigra tissues were fixed in 10% neutral buffered formalin for 24 h. Thereafter, specimens were thoroughly washed under running water, dehydrated in graded ethanol, cleared in xylol, and finally embedded in paraffin wax. Subsequently, microtome sections five microns thick were obtained using a manual rotatory microtome (SLEE rotatory microtome, CUT, 4062, Germany) and mounted on glass slides. A section set was prepared for histological investigation using the conventional techniques of Hematoxylin & Eosin staining (Drury & Wallington, 1980) [38]. Using a digital camera coupled to the microscope, representative images of both areas were taken, five fields were assessed and the mean count of normal and altered neurons per field was calculated. Livre and kidney histological alteration were assessed to study extract toxicity in the control, DMSO, and extract-treated group (S1 Table).

#### Tyrosine hydroxylase and alpha-synuclein immunostaining

For tyrosine hydroxylase and alpha-synuclein immune staining, by using positively charged slides, the paraffinized serial paraffin sections were cut, dewaxed, and hydrated. Antigen retrieval was completed by heating the slides with citrate buffer for 15 minutes at pH 6, incubated overnight with Tyrosine hydroxylase primary antibody (rabbit monoclonal, clone EP1532Y, #ab137869, Abcam, USA) at a concentration of 1: 100 on one slide. Alpha-synuclein primary antibody (rabbit monoclonal, clone EPR20535, ab212184, Abcam, USA) was used in the other one. The next day, sections with peroxidase (HRP) conjugated secondary antibody were incubated For (mention the incubation period and the washing required to remove excessive antibodies). After all, signals were visualized by diaminobenzidine (DAB) followed by Mayer’s hematoxylin counterstaining. Positive staining was seen as brown color.

In sections, stained with tyrosine hydroxylase, substantia nigra was identified and several images were captured at ×200 power of non-overlapping fields. Using image J software, positively stained cells were counted in the substantia nigra and the optical density of terminal staining was measured in the striatum [39]. Different images were taken at ×400 magnifications, and then an average measurement was obtained for each rat [40].

Three non-overlapping fields were photographed and examined in alpha synuclein-stained sections where positive neurons were counted in substantia nigra and striatum separately per ×400 field.

### Gene expression analysis

Total RNA was extracted from cells using the TriRNA Pure Kit with DNase, following the manufacturer’s protocol. For each sample, one microgram of total RNA was used to synthesize cDNA with the TOPscript™ cDNA Synthesis Kit. The expression levels of various coding genes were analyzed using quantitative real-time PCR assays on a SimpliAmp Thermal Cycler (ThermoFisher Scientific, USA). The genes tested included dopa decarboxylase (Ddc) with forward primer sequence CCGCTTCAGAGACCCAAAGT and reverse primer sequence CACGGCCACACAAAGAACAG, tyrosine hydroxylase (Th) with forward primer sequence CTGTCACGTCCCCAAGGTTC and reverse primer sequence TTACAGCCCGAGACAAGGAG, and beta-actin (Actb) with forward primer sequence CACCCGCGAGTACAACCTTC and reverse primer sequence GGATGCCTCTCTTGCTCTGG. For each gene, a master mix was prepared using Maxima SYBR Green qPCR Master Mix (2×) according to the manufacturer’s instructions. Relative gene expression levels between samples were calculated using the threshold cycle (CT) values, employing the ΔΔCT method.

## Results

### *P*. *lividus* gonadal extract administration ameliorates rotenone-induced behavioral abnormalities and motor dysfunction in rats

**Fig 1:**
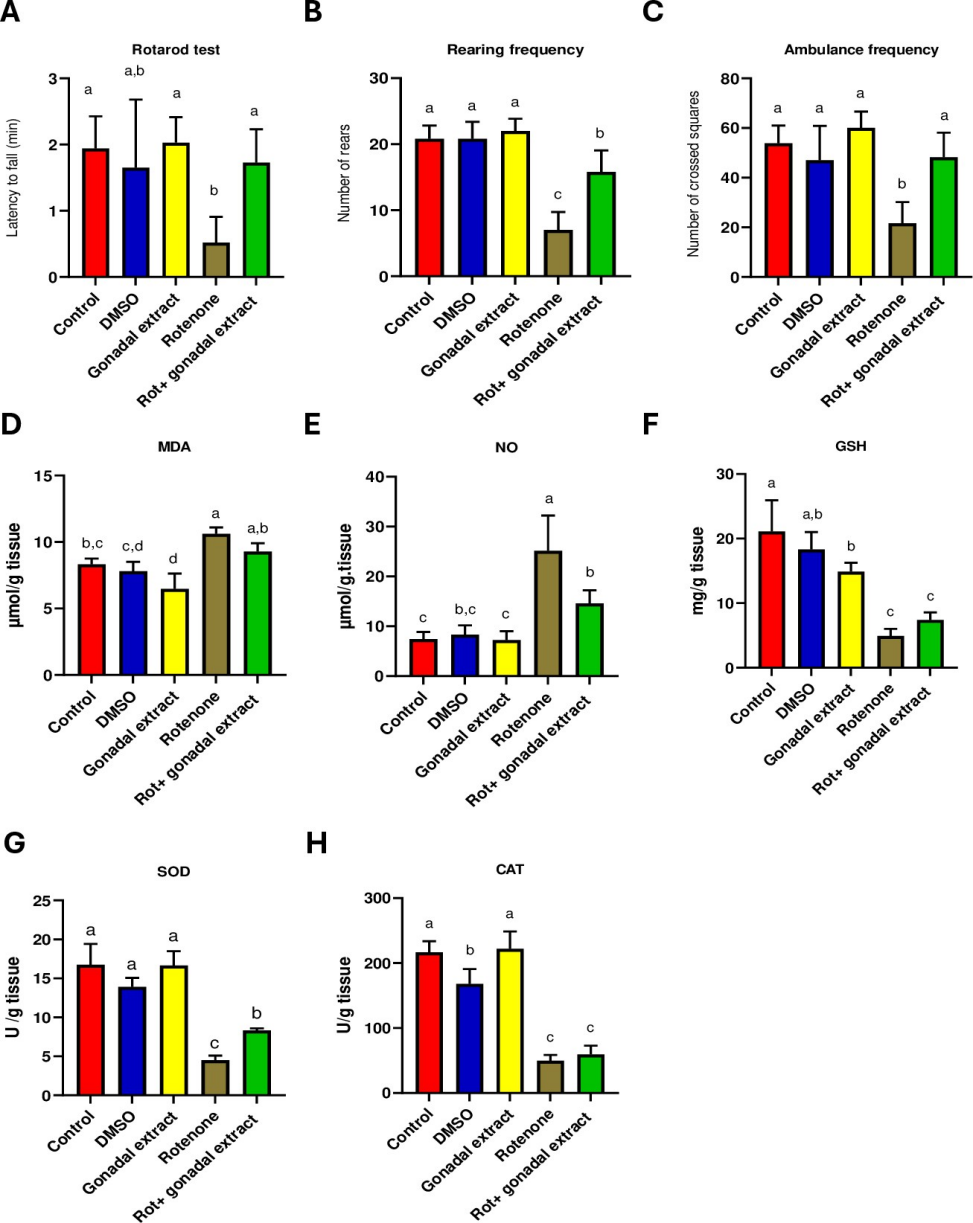
(A-C): Effect of *P*. *lividus* gonadal extract on the behavioral alternation induced by rotenone in rats. (A) Rotarod test scores. (B&C) Open field test, measuring rearing frequency & Ambulance frequency. (D-H): Effect of *P*. *lividus* gonadal extract on rotenone-induced oxidative impairments in nigrostriatal tissue of rats. Oxidative stress markers (MDA, NO, and GSH) and antioxidant enzymes (SOD and CAT) were determined. All the data were analyzed using one-way ANOVA followed by Tukey Pairwise Comparisons. Values are expressed as mean ± SEM; n = 5 rats for each group. Different superscripts on the columns are significantly different at p≤0.05.

To evaluate the impact of rotenone-induced neurotoxicity, we administered 2mg/kg of rotenone subcutaneously into Wistar male rats. Compared to DMSO-treated control group, rotenone administration exhibited a significant motor deficiency in the rats, as evidenced by a reduced latency time on the rotarod test (**Fig 1A**). Conversely, rats treated with both rotenone + *P*. *lividus* gonadal extract showed a significant increase in rotarod latency compared to the rotenone-only group (**Fig 1A**). Additionally, locomotor activity, assessed through rearing and ambulation frequency in the open field test, was analyzed for both control and experimental rats (**Fig 1B & 1C**). Rotenone administration led to a significant decrease in both rearing and ambulation frequencies. However, co-administration of *P*. *lividus* gonadal extract + rotenone significantly improved these locomotor parameters, as demonstrated by increased rearing and ambulation frequencies (**Fig 1B & 1C**).

### Effect of *P*. *lividus* gonadal extract on rotenone-induced oxidative impairments

The oxidative status of the substantia nigra and striatum was assessed by measuring MDA, NO, and GSH levels as well as the activity of SOD and CAT. As shown in (**Fig 1D–1H**), the administration of Rot, significantly elevated MDA and NO levels (**Fig 1D & 1E**), while GSH level and SOD and CAT activities were markedly declined compared to the control groups (**Fig 1F–1H**). Combined treatment of *P*. *lividus* gonadal extract and Rot showed a significant decline in NO level, slightly decreased MDA level, slightly increased GSH and CAT levels and significantly increased SOD activity compared to the Rot-treated rats (**Fig 1D–1H**).

### *P*. *lividus* gonadal extract attenuates rotenone-induced neuroinflammation and restores dopamine levels in nigrostriatal tissues

**Fig 2:**
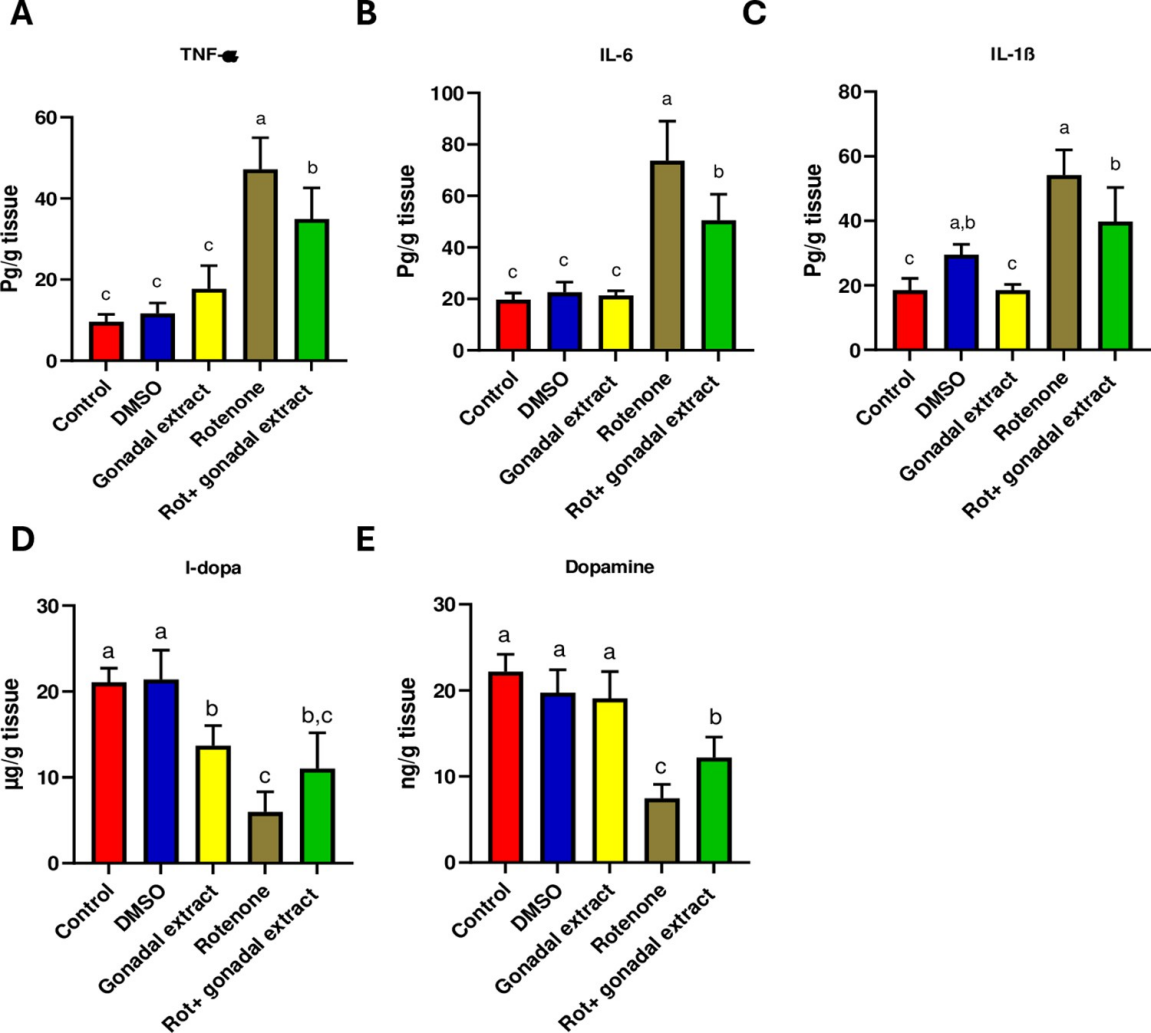
Effect of *P*. *lividus* gonadal extract on rotenone-induced neuroinflammation and its effect on levels of L-dopa and dopamine in nigrostriatal tissue of male rats. Proinflammatory cytokines (TNF-α, IL-6 and IL-1β) levels were measured. All the data were analyzed using one-way ANOVA Tukey Pairwise Comparisons. Values are expressed as mean ± SEM; n = 5 rats for each group. Different superscripts on the columns are significantly different at p≤0.05.

Neuroinflammation plays a crucial role in the pathophysiology of PD [41]. Therefore, we sought to investigate the potential effect of the gonadal extract on pro-inflammatory markers. Notably, the one-way ANOVA test revealed a significant difference in the levels of TNF-α, IL-6, and IL-1β among different experimental groups. As depicted in (**Fig 2A–2C**), ELISA analysis indicated that the levels of these pro-inflammatory cytokines were significantly elevated in rats treated with rotenone compared to the control group. Nevertheless, the administration of *P*. *lividus* gonadal extract significantly inhibited this increase in cytokine levels, indicating an anti-inflammatory effect of the extract (**Fig 2A–2C**). Furthermore, upon rotenone treatment, the levels of DA and L- DOPA in the substantia nigra and striatum were significantly decreased compared to controls. Administration of the gonadal extract significantly improved the levels of DA and L-DOPA in the tissues of both brain regions as compared to Rot-treated rats (**Fig 2D & 2E**).

### Effect of *P*. *lividus* gonadal extract on the histology of substantia nigra

**Fig 3:**
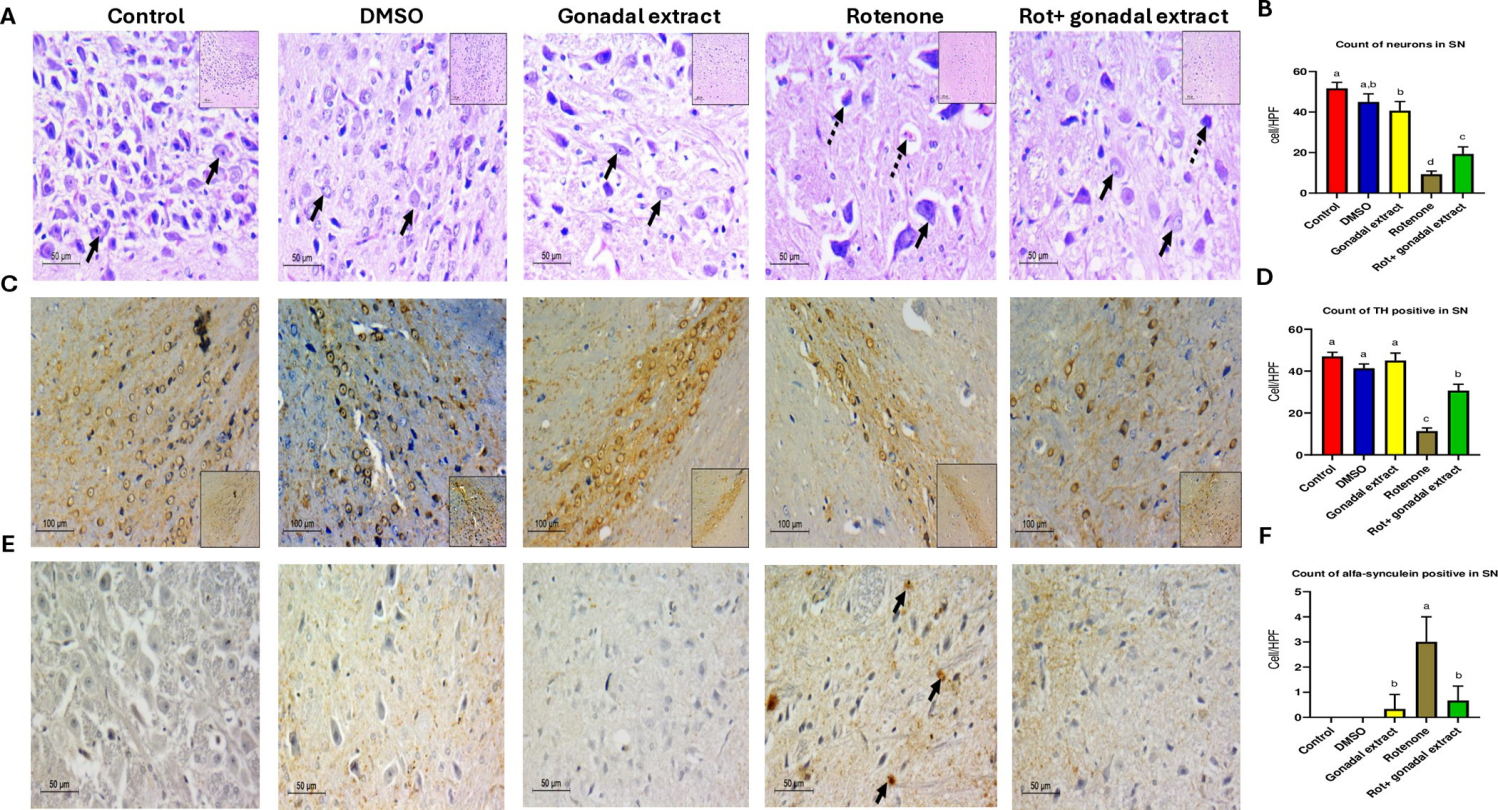
A) Pathologic assessment of H&E-stained sections of substantia nigra in different studied groups: control, DSMO, and gonadal extract groups show a compact cellular substania nigra in low power, high power shows viable neurons with large, rounded nuclei with open chromatin and a nucleolus (arrows). The Rotenone group shows less cellular loose substantia nigra, and high power shows degenerated neurons with dark stained nuclei (Dashed arrows) with few viable ones (arrows). The gonadal extract treated rotenone group shows restoration of neurons in SN, high power shows viable neurons (arrows), and fewer degenerated ones (dashed arrows). (H&E, low power x200, scale bar = 100 microns, high power x400, scale bar = 50 microns). B) Count of neurons in substantia nigra. C) Pathologic assessment of tyrosine hydroxylase-stained sections of substantia nigra in different studied groups highlighting positive neurons in each group. Many dopaminergic neurons are seen in deep brown backgrounds in control, DSMO, and gonadal extract groups. The Rotenone group shows a markedly diminished number of positive staining neurons. The gonadal extract-treated rotenone group shows increased expression (IHC, x200, scale bar 100 microns, inset x100). D) Count of TH-positive neurons in substantia nigra. E) Pathologic assessment of α- synuclein stained sections of substantia nigra in different studied groups highlighting positive neurons in each group. Negative staining of neurons is seen in control, DSMO, and gonadal extract groups. The Rotenone group shows multiple positive α—synuclein neurons (arrows) and the gonadal extract-treated rotenone group shows decreased expression. (IHC, x400, scale bar 50 microns). F) Count of α-synuclein positive in substantia nigra. All the data were analyzed using one-way ANOVA followed by Tukey Pairwise Comparisons. Values are expressed as mean ± SEM; n = 3 rats for each group. Different superscripts on the columns are significantly different at p≤0.05.

As shown in **Fig 3A**, the substantia nigra (SN) of the control group consisted of large neurons with deep basophilic granular cytoplasm and large vesicular nuclei with prominent nucleoli. The neuropil was intact and thick. A similar histoarchitecture was observed in the DMSO and gonadal extract groups, displaying viable neurons within a dense fibrillary background. In contrast, the Rot group exhibited significant pathological changes. The neurons in the substantia nigra were dispersed and widely spaced, showing signs of neurodegeneration such as vacuolated neuropil, dark-stained eccentric nuclei, and pale cytoplasm with a lower cell count compared to other experimental groups. In the group treated with rotenone + *P*. *lividus* gonadal extract, a restoration of neural histology was noted, the mean count of viable neurons increased significantly, harboring intact vesicular nuclei and thick neuropils. The count of neurons in the SN per ×400 field is presented in **Fig 3B**.

Rotenone administration resulted in a significant downregulation of tyrosine hydroxylase (TH) expression (**Fig 3C**) and a high accumulation of α-synuclein-positive bodies (**Fig 3E**) in the large dopaminergic neurons of the substantia nigra compared to the control groups. However, in animals co-treated with rotenone and gonadal extract, there was an improvement in TH expression levels and a reduction in intracellular α-synuclein accumulation compared to the rotenone-only group. The count of TH-positive neurons in the substantia nigra and the count of α-synuclein-positive bodies in the substantia nigra, both showing significant differences, are presented in **Fig 3D and 3F**. Full stack images can be accessed in **S1–S3 Figs**.

### Effect of *P*. *lividus* gonadal extract on the histology of striatum

**Fig 4:**
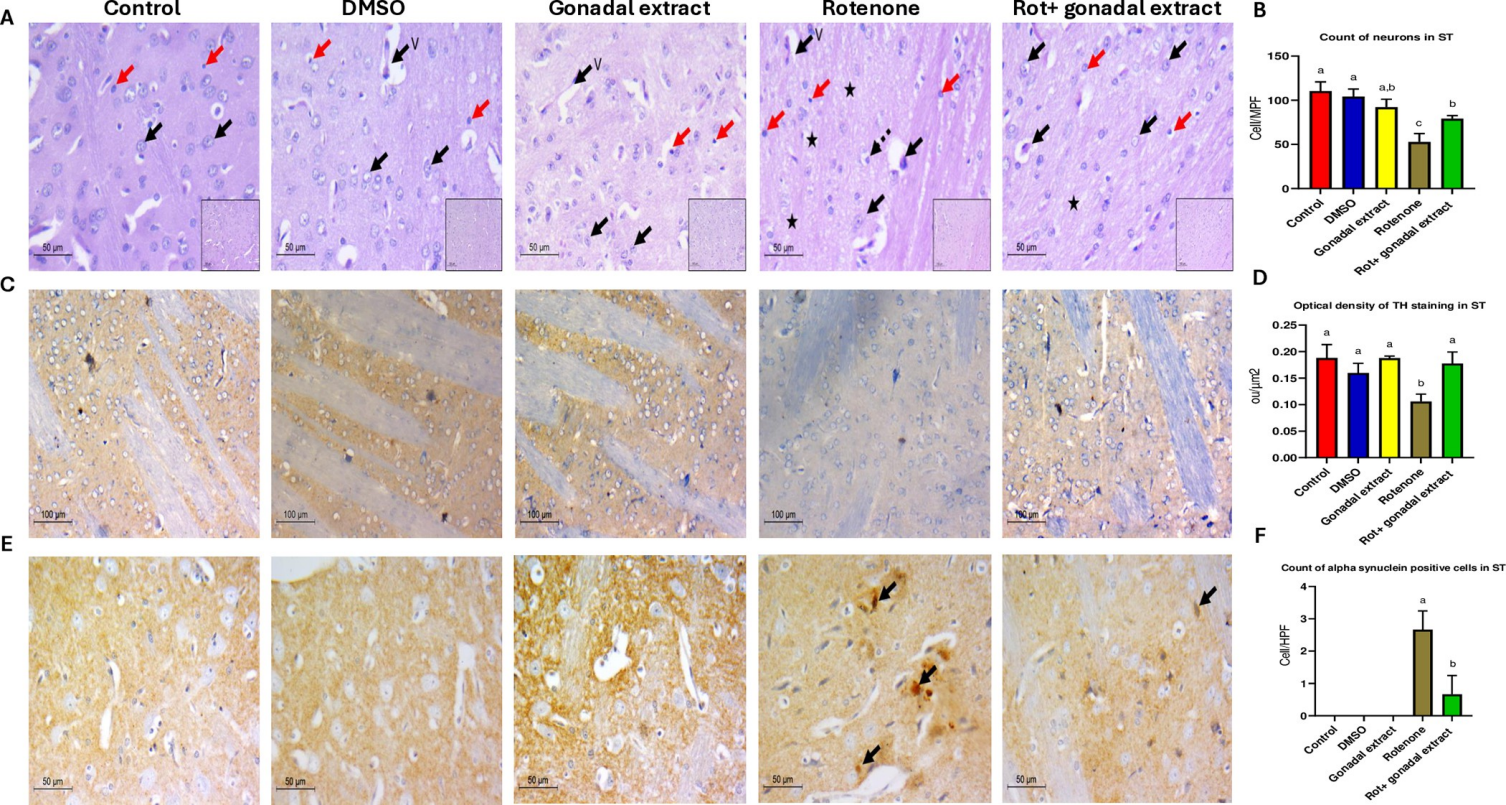
A) Pathologic assessment of H&E-stained sections of the striatum in different studied groups: control, DSMO, and gonadal extract groups show a cellular striatum, high power shows viable neurons with large, rounded nuclei with open chromatin and a nucleolus (black arrows), few microglial cells (red arrows) and thin capillaries are seen (v). The rotenone group shows evident disturbed architecture and hypocellularity, high power shows few viable neurons (black arrows), and multiple degenerated neurons with dark stained nuclei (Dashed arrows), the background shows vacuolated neuropil (star) and increased microglial cells (red arrows). The gonadal extract-treated rotenone group shows improvement of architecture of the striatum, high power shows viable neurons (arrows) with residual focal neuropil vacuolation (star), and few microglial cells are seen (red arrows) (H&E, low power x200, scale bar = 100 microns, high power x400, scale bar = 50 microns). B) Count of neurons in the striatum in different groups. C) Pathologic assessment of tyrosine hydroxylase-stained sections of the striatum in different studied groups highlighting positive dopaminergic terminals in each group. A high-density deep brown background is seen in the control, DSMO, and gonadal extract groups. The Rotenone group shows decreased staining density, while the gonadal extract-treated rotenone group shows increased staining. (IHC, x200, scale bar 100 microns, inset x100). D) Optical density of TH staining in the striatum. E) Pathologic assessment of α- -synuclein stained sections of striatum in different studied groups highlighting positive neurons in each group. Negative staining of neurons is seen in control, DSMO, and gonadal extract groups. The Rotenone group shows multiple positive α—synuclein neurons (arrows), while the gonadal extract-treated rotenone group shows decreased expression. (IHC, x400, scale bar 50 microns). F) Count of α—synuclein positive cells in the striatum. All the data were analyzed using one-way ANOVA followed by Tukey Pairwise Comparisons. Values are expressed as mean ± SEM; n = 3 rats for each group. Different superscripts on the columns are significantly different at p≤0.05.

The striatum, as shown in **Fig 4A**, is comprised of large neurons with open-face chromatin and small eccentric nucleoli, along with a few thin capillaries and microglial cells. This normal histoarchitecture was maintained in the control, DMSO, and gonadal extract groups. However, the Rot group displayed marked neuronal hypocellularity, indicated by a decreased count of viable neurons, vacuolated pale neuropil in the background, increased microglial cells, and perivascular edema. These pathological changes were reversed in the brains treated with *P*. *lividus* gonadal extract, as evidenced by an increased count of viable neurons (**Fig 4B**).

Rotenone administration resulted in a significant downregulation of tyrosine hydroxylase (TH) expression (**Fig 4C**) and a high accumulation of α-synuclein-positive bodies (**Fig 4E**) in the large dopaminergic neurons of the striatum compared to the control groups. However, in animals co-treated with rotenone and gonadal extract, there was an improvement in TH expression levels and a reduction in intracellular α-synuclein accumulation compared to the rotenone-only group. The optical density of TH staining in the striatum and the count of α-synuclein-positive cells in the striatum demonstrated significant differences among the investigated groups (**Fig 4D and 4F**). Full stack images can be accessed in **S4–S6 Figs**. Finally, to role out any potential toxicity of the sea urchin extract, we analyzed function enzymes and histology of the liver and kidney in the control, DMSO, and gonad extract group. Results show no toxicity of the extract **S7 and S8 Figs**.

### *P*. *lividus* gonadal extract significantly impacts *dopa decarboxylase* expression levels

**Fig 5:**
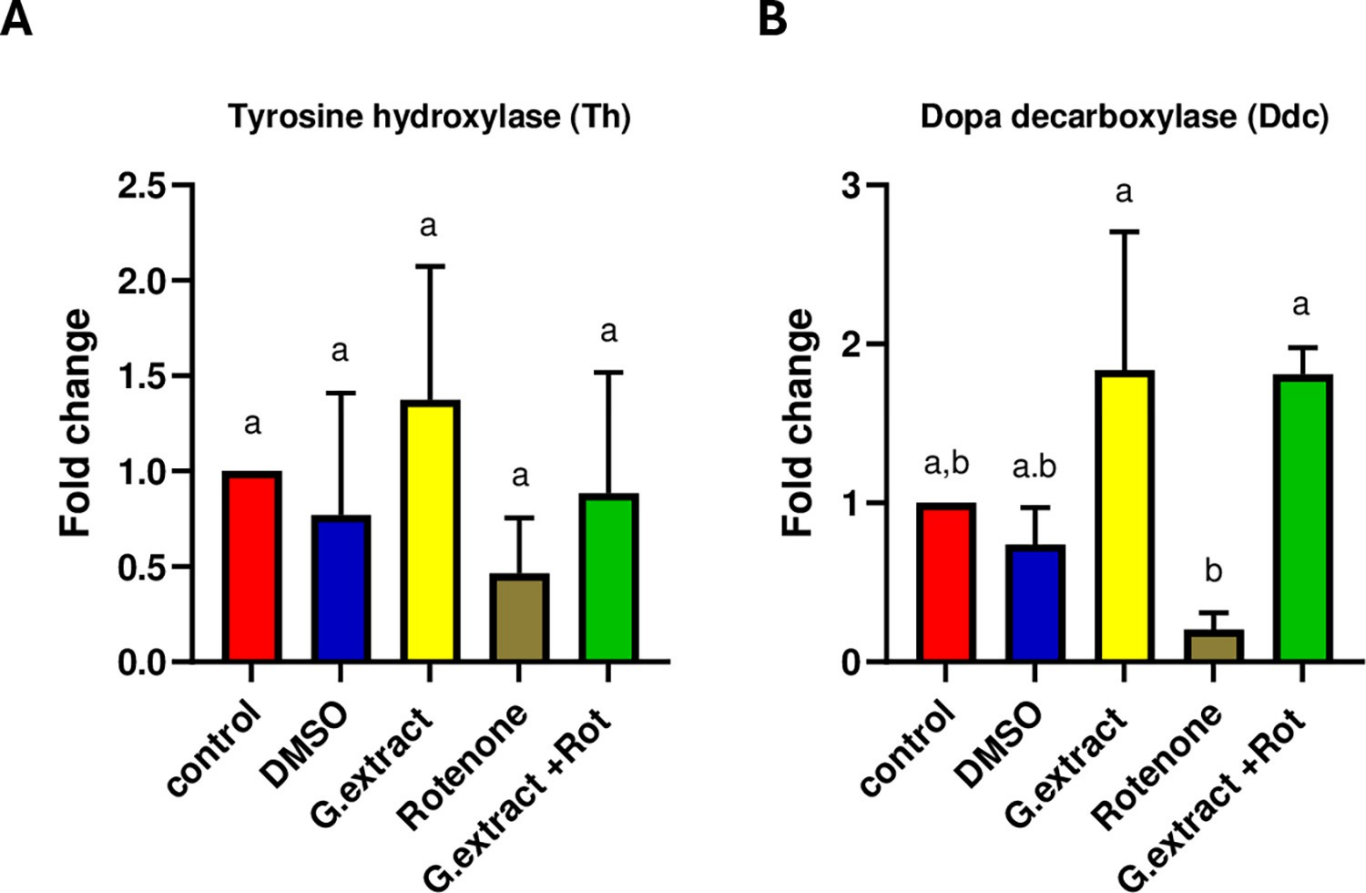
Gene expression of tyrosine hydroxylase (Th) and dopa decarboxylase (Ddc) after treatment with P. *lividus gonadal* extract and RT-qPCR were performed in triplicates. All the data were analyzed using one-way ANOVA followed by Tukey Pairwise Comparisons. Values are expressed as mean ± SEM. Different superscripts on the columns are significantly different at p≤0.05.

The effect of the *P*. *lividus* gonadal extract on *TH* expression patterns and *dopa decarboxylase (Ddc)*, key genes in dopamine synthesis in neurons [42], was assessed at the transcriptional level utilizing real-time quantitative PCR assays. As illustrated in **Fig 5A**, *Th* expression was upregulated in the *P*. *lividus* gonadal extract group, downregulated in the rotenone group, and restored in the group receiving combined treatment. However, these changes in *TH* expression were not statistically significant. Conversely, a similar expression pattern was observed for *Ddc*, but the changes in *Ddc* expression levels were statistically significant (**Fig 5B**).

## Discussion

Parkinson’s disease is a debilitating neurodegenerative disorder caused by the impairment of the nigrostriatal dopaminergic pathway; however, the molecular and cellular underlying mechanisms are still unclear [43]. Previous studies have indicated that risk factors, including neuroinflammation, mitochondrial malfunction [44,45], and oxidative stress [46], are involved in the development and progression of PD. These elements can trigger cellular autophagy or apoptosis [47]. Our study aimed to investigate the neuroprotective effect of *P*. *lividus* gonadal extract on a rat model of Parkinson’s disease induced by Rot, which showed promising findings in alleviating disease progression and pathologies.

In the current study, administration of a low dose of Rot 2 mg/kg for six weeks limited the motor abilities of rats. The observed short latency time, decreased rearing, and ambulance frequency demonstrate diminished exploratory and locomotor activities in Rot-treated rats. These motor impairments were previously observed in PD animal models [48,49] and suggest that Rot administration induces features of Parkinsonism [28]. Gonadal extract treatment caused increasing latency time in the rotarod test and increased rearing and ambulance in the open field test. The current behavior test results pattern was also found in a previous study [50], which found that metformin had neuroprotective properties and markedly enhanced the rotarod performance of metformin-treated mice, increased the number of rearing, and enhanced the overall locomotor behavior. These results suggested an improvement in muscular activity and locomotion compared to rot-treated animals [51].

Additionally, our study demonstrated that rotenone administration exacerbated nigrostriatal oxidative damage, as evidenced by elevated levels of MDA and NO, indicative of increased ROS production. Conversely, endogenous antioxidants, including GSH, SOD, and CAT, were reduced following rotenone treatment. Previous studies have also shown that rotenone administration significantly increases nigrostriatal MDA and NO levels while decreasing GSH levels and the activities of both SOD and CAT [28,52]. In contrast, supplementation with *P*. *lividus* gonadal extract prevented the rotenone-induced alterations in the redox status of nigrostriatal tissue. This was evidenced by the reduction of MDA and NO formation, along with an enhancement in the antioxidant activity. These findings underscore the promising neuroprotective and antioxidative properties of *P*. *lividus* gonadal extract. In this context, the gonads of *P*. *lividus* contain various carotenoids such as astaxanthins [18], known for their antioxidant and neuroprotective activities and ability to cross the blood-brain barrier [53]. Recently, Khalil et al. [22] reported that sea urchin extracts exhibit strong neuroprotective effects and prevent cisplatin-induced oxidative stress in rats. Antioxidant activity of different natural extracts was found to have a significant role in the progression of PD, like *Mucuna pruriens* (Mp), which possesses therapeutic activity against PD, as it has Ursolic acid (UA) which is a powerful bioactive component that has anti-inflammatory and anti-oxidative activity in toxin-induced PD model [54].

Dopaminergic neurodegeneration *was* attenuated in the PD mouse model after treatment with the *Withania somnifera* (Ws) root extract due to the ability of Ws extract to inhibit the oxidative stress occurring in nigrostriatal tissues [55].

At a molecular level, NF-κB activation is needed to produce pro-inflammatory cytokines (TNF-α, IL-1β, and IL-6) by microglia [56], producing pro-inflammatory cytokines activates microglia which mainly contributes to neurodegeneration. Mediators with anti-inflammatory activity could act against PD development. The expression levels of the pro-inflammatory cytokines TNF-α, IL-6, and IL-1β were significantly elevated in rats treated with rotenone, indicating that rotenone induces pro-inflammatory responses. The oxidative stress observed in our study likely stimulated the induction of these cytokines. Previous studies [57–59] have shown that rotenone administration results in microglial activation, the release of neuroinflammatory cytokines, and exacerbation of oxidative and nitrosative stress, accompanied by a reduction in antioxidant potential. Conversely, a combined treatment with *P*. *lividus* gonadal extract prevented the rotenone-induced increase of the pro-inflammatory cytokines IL-1β, IL-6, and TNF-α levels. Reducing the levels of these cytokines is a positive indicator of mitigating neuroinflammation in PD models, as supported by earlier research [60,61]. Of note, we have previously characterized the *P*. *lividus* gonadal extract and found it to contain high levels of bioactive compounds, particularly carotenoids, which exhibit anti-inflammatory and antioxidant properties [18]. The anti-neuroinflammatory properties of carotenoids have been demonstrated both in vitro and in vivo [62]. Earlier reports indicated that organic extracts from the gonads of *Stomopneustes variolaris* inhibit COX-2 and 5-lipoxygenase, two enzyme families critical to the inflammatory process [63]. Additionally, several studies [64] have shown that astaxanthin acts as a multi-target drug by inhibiting mediators of neuroinflammation and oxidative stress pathways, which are crucial in preventing the progression of PD. Treatment with astaxanthin has been shown to reduce the levels of TNF-α, IL-1β, and IL-6, exerting a significant anti-inflammatory effect [65–67]. Furthermore, astaxanthin, echinenone, β-carotene, and lycopene have been found to suppress ROS production [68]. Like astaxanthin, it was found that metformin (used as a first-line therapy for type 2 diabetes mellitus) can decrease the cellular level of NF-κB which leads to decreasing the cellular levels of TNF-α, IL-1β, and IL-6 [69].

Dopamine is synthesized in the neuronal cytoplasm through the action of tyrosine hydroxylase (TH), which converts tyrosine into L-DOPA. Then, L-DOPA is converted into dopamine by dopa decarboxylase (Ddc) [70,71]. Consistent with previous reports, the current study observed a significant reduction in the levels of DA and L-DOPA following rotenone administration. This decline can be attributed to the damage inflicted on dopaminergic neurons [49], a finding corroborated by our histopathological analysis. Supporting our results, earlier studies [72–74] have demonstrated that rotenone administration induces neuronal cell loss, apoptotic changes, and neurodegeneration in the substantia nigra and striatum of rats and mice. However, our study found that pre-treatment with gonadal extract restored neural histology and increased DA and L-DOPA levels in rats exposed to rotenone. Several studies have shown that marine natural products significantly enhance brain neurotransmitter levels. Lycopene, an aliphatic hydrocarbon carotenoid and one of the active ingredients in sea urchin extract, has been found effective in reversing neurochemical defects, apoptosis, and physiological abnormalities in mice with Parkinson’s disease [75]. To evidence our results further, we investigated the expression levels of *TH* and *Ddc* using the RT-PCR. Our findings revealed that *TH* and *Ddc* expression levels were significantly downregulated in the nigrostriatal tissue of male rats treated with rotenone consistent with previous in vivo and in vitro studies [28,76]. However, pre-treatment with *P*. *lividus* gonadal extract significantly increased *TH* and *Ddc* levels in rotenone-exposed rats. Although the RNA expression levels of *TH*, assessed by RT-qPCR, did not demonstrate a statistically significant difference, immunohistochemistry assessment of TH exhibited a remarkable difference among the investigated groups. These observations reflect that the gonadal extract does not modulate the transcriptional activity of TH; however, it may affect downstream translational or post-translational modifications that alter TH protein stability. It is well established that changes in *TH* mRNA levels do not always correlate with TH protein expression, TH enzymatic activity, or catecholamine function [77]. Several factors, including feedback inhibition and catecholamine inactivation, can influence these discrepancies [78]. So, increasing dopamine concentration initiated the feedback inhibition mechanism leading to a decrease in the mRNA levels. Similarly, carotenoids have been shown to play a prophylactic role in Parkinson’s disease by suppressing mitochondrial dysfunction and restoring TH activity [79]. Additionally, enhancing Ddc activity has been proposed as a potential treatment for Parkinson’s disease [80]. The ability of *P*. *lividus* gonadal extract to improve the activity and expression of TH and Ddc highlights its potential as a promising neuroprotective or therapeutic agent.

The current immunohistochemical results revealed a significant decline in TH levels and numerous α-synuclein-positive aggregates in the substantia nigra (SN) and striatum of rats treated with rotenone. Previous studies have shown that rats administered rotenone exhibit a notable decrease in TH immune positive neurons in the SN (21%) and striatum (10%) [81]. Additionally, significant decreases in TH expression [82] and increases in α-synuclein [83] have been observed in the brains of mice treated with rotenone. Overexpression of α-synuclein induced by rotenone has been documented in both the striatum and SN [84]. α-synuclein accumulation produces oxidative stress, various oxygen-free radicals, such as superoxide (O2–), ROS, and cytotoxic factors such as IL-1β, TNF-α, and other neurotoxic substances, increased significantly leading to continuous injury and DA neuron apoptosis [85]. Rotenone was found to inhibit complex I, and induce serine phosphorylation of α-syn, resulting in the cytoplasmic protein aggregates formation [86]. However, co-treatment with rotenone and *P*. *lividus* gonadal extract resulted in increased TH level and decreased intracellular α-synuclein aggregations in the SN and striatum, indicating the neuroprotective effect of the sea urchin gonadal extract. Reducing α-synuclein accumulation has been shown to provide neuroprotection against Parkinson’s disease [87]. The control of inflammation and α-syn aggregation and propagation are potential targets for disease-modifying treatments in PD [88]. For instance, two compounds (HSEA-P1 and P2) purified from *Holothuria scabra* significantly diminished α-synuclein accumulation and protected dopaminergic neurons from α-synuclein toxicity in a *C*. *elegans* model [89]. Palmitic acid, decanoic acid, and stearic acid were detected in the *P*. *lividus* gonadal extract. Palmitic acid, derived from *Holothuria leucospilota*, has been shown to attenuate the loss of dopaminergic neurons, improve dopamine-dependent behaviors, and decrease α-synuclein aggregation, thus exhibiting anti-Parkinson effects [90]. Similarly, decanoic acid, isolated from the same sea cucumber species, has demonstrated anti-Parkinson effects in *C*. *elegans* PD models [91]. Additionally, stearic acid has shown promising effects on locomotion activity [92]. In conclusion, as alpha-synuclein plays a crucial role in Parkinson’s pathology, future studies should focus on its cellular and molecular modulation, as well as the long-term effects of *P*. *lividus* gonadal extract upon treating experimental animal models for longer time periods.

Results showed that the count of TH positive in Substania nigra and Striatum was increased after *P*. *lividus* gonadal extract treatment, and there was no significant change in the RT-PCR results (mRNA levels). It is known that TH mRNA level changes do not necessarily correspond to TH protein, TH activity, or catecholamine function [77], this means that the modulation of tyrosine hydroxylase activity can be achieved by nearly every documented form of regulation. TH has a direct role in the pathogenesis of PD, especially through oxidative stress and pro-inflammatory mechanisms [93]. *P*. *lividus* gonadal extract was able to affect the proinflammatory cytokines and exert antioxidant activity against PD induced in rats, supporting its ability to modulate the transcriptional modifications of TH. The presence of NO can modify TH resulting in nitration of tyrosine residues and the glutathionylation of cysteine residues. Also, the TH enzyme is inhibited in a feedback manner by the catecholamine neurotransmitters [94]. As the oxidative stress and NO were reduced by *P*. *lividus* gonadal extract treatment, we supposed that the extract can affect the protein-protein interactions that modulate TH activity confirming its role in post-translational modifications of tyrosine hydroxylase.

The neurodegenerative-related genes share a 90% similarity between humans and rats including Parkinson’s disease genes [95]. This implies the conserved molecular pathways between rats and humans. Knowing that *P*. *lividus* is a well-accepted dietary marine animal and the similarity of neurodevelopmental genes between rats and humans we postulate the applicability of our extract as a single or combinatory therapeutic regime to treat Parkinson’s disease in humans. Therefore, our findings briefly indicate that *P*. *lividus* gonadal extract exhibits a promising neuroprotective effect against rotenone-induced neurotoxicity. These protective effects are likely mediated through the extract’s antioxidant, anti-inflammatory, and anti-apoptotic properties. Rotenone has been used to induce Parkinson’s Disease in different animal models [96], such as drosophila [97,98], zebrafish [99], and mouse [100]. This could raise the possibility of applying sea urchin gonad extract as a neuroprotective agent in those model animals. We recommend further attention to *P*. *lividus* gonads as a valuable dietary supplement with potential benefits for health improvement and protection against neurodegenerative diseases such as Parkinson’s disease.

## Supporting information

S1 Fig Pathologic assessment of H&E-stained sections of substantia nigra different studied groups: A) normal, B) DSMO, C) Gonadal extract groups show a compact cellular substania nigra in low power. High power shows viable neurons with large, rounded nuclei with open chromatin and a nucleolus (arrows). D) rotenone group showing less cellular loose substantia nigra. High power show degenerated neurons with dark stained nuclei. (Dashed arrow) with few viable ones (arrow). E) gonadal extract treated rotenone group shows restoration of neurons in SN. High power shows viable neurons (arrows) and fewer degenerated ones (dashed arrows). (H&E, low power x200, scale bar = 100 microns, high power x400, scale bar = 50 microns).
(PPTX)

S2 Fig Pathologic assessment of tyrosine hydroxylase stained sections of substantia nigra in different studied groups highlighting positive neurons in each group. large number of dopaminergic neurons are seen in deep brown background in A) normal, B) DSMO, C) Gonadal extract groups. D) rotenone group showing markedly diminished number E) gonadal extract treated rotenone group shows restoration of neurons in SN. (IHC, x200, scale bar 100 microns, inset x100).
(PPTX)

S3 Fig Pathologic assessment of alpha synuclein stained sections of substantia nigra in different studied groups highlighting positive neurons in each group. negative staining of neurons is seen in A) normal, B) DSMO, C) Gonadal extract groups. D) rotenone group showing multiple positive alpha synuclein neurons (arrows) E) gonadal extract treated rotenone group decrease expression. (IHC, x400, scale bar 50 microns).
(PPTX)

S4 Fig Pathologic assessment of H&E-stained sections of striatum different studied groups: A) normal, B) DSMO, C) Gonadal extract groups show a cellular striatum. High power shows viable neurons with large, rounded nuclei with open chromatin and a nucleolus (black arrows). few microglial cells (red arrows) and thin capillaries are seen (v) D) rotenone group showing evident disturbed architecture and hypocellularity. High power show few viable neurons (black arrows), multiple degenerated neurons with dark stained nuclei (Dashed arrow). The background shows vacuolated neuropil (star) and increased microglial cells (red arrows) E) gonadal extract treated rotenone group shows improvement of architecture of striatum. High power shows viable neurons (arrows) with residual focal neuropil vacuolation (star). Few microglial cells are seen (red arrows) (H&E, low power x200, scale bar = 100 microns, high power x400, scale bar = 50 microns).
(PPTX)

S5 Fig Pathologic assessment of alpha synuclein stained sections of striatum in different studied groups highlighting positive neurons in each group. negative staining of neurons is seen in A) normal, B) DSMO, C) Gonadal extract groups. D) rotenone group showing multiple positive alpha synuclein neurons (arrows) E) gonadal extract treated rotenone group decrease expression. (IHC, x400, scale bar 50 microns).
(PPTX)

S6 Fig Pathologic assessment of tyrosine hydroxylase-stained sections of striatum in different studied groups highlighting positive dopaminergic terminal in each group. high density deep brown background is seen in A) normal, B) DSMO, C) Gonadal extract groups. D) rotenone group showing decreased staining density. E) gonadal extract treated rotenone group shows increased staining. (IHC, x200, scale bar 100 microns, inset x100).
(PPTX)

S7 Fig Effect of *P*. *lividus* gonadal extract on the toxicity in rats, GOT (AST) Glutamic—Oxaloacetic Transaminase, Alkaline phosphatase (ALP), GPT (ALT) Glutamic–Pyruvic Transaminase, Urea, uric acid, and creatinine levels were detected. All the data were analyzed using one-way ANOVA followed by Tukey Pairwise Comparisons. Values are expressed as mean ± SE; n = 5 rats for each group. Different superscripts on the columns are significantly different at p≤0.05.
(PPTX)

S8 Fig H&E stained sections of liver (first column, x100) and kidney (second column, x200) of different studied groups. **A, B** control**, C, D** DMSO, **E, F** *P*. *lividus* gonadal extract. No pathologic changes were detected in both organs in all studied rats.
(PPTX)

S1 Table List of liver and kidney function tests used to ass toxicity of *P*. *lividus* gonad extract. (DOCX)
