# Supplementary material for: Paracentrotus lividus sea urchin gonadal extract mitigates neurotoxicity and inflammatory signaling in a rat model of Parkinson’s disease
Source: PLoS One. 2024 Dec 18;19(12):e0315858. doi: 10.1371/journal.pone.0315858 (PMC11654954; doi:10.1371/journal.pone.0315858)
Supplement: S1 Fig — Pathologic assessment of H&E-stained sections of substantia nigra different studied groups: A) normal, B) DSMO, C) Gonadal extract groups show a compact cellular substania nigra in low power. High power shows viable neurons with large, rounded nuclei with open chromatin and a nucleolus (arrows). D) rotenone group showing less cellular loose substantia nigra. High power show degenerated neurons with dark stained nuclei. (Dashed arrow) with few viable ones (arrow). E) gonadal extract treated rotenone group shows restoration of neurons in SN. High power shows viable neurons (arrows) and fewer degenerated ones (dashed arrows). (H&E, low power x200, scale bar = 100 microns, high power x400, scale bar = 50 microns). (PPTX) [file pone.0315858.s001.pptx]

## Slide 1
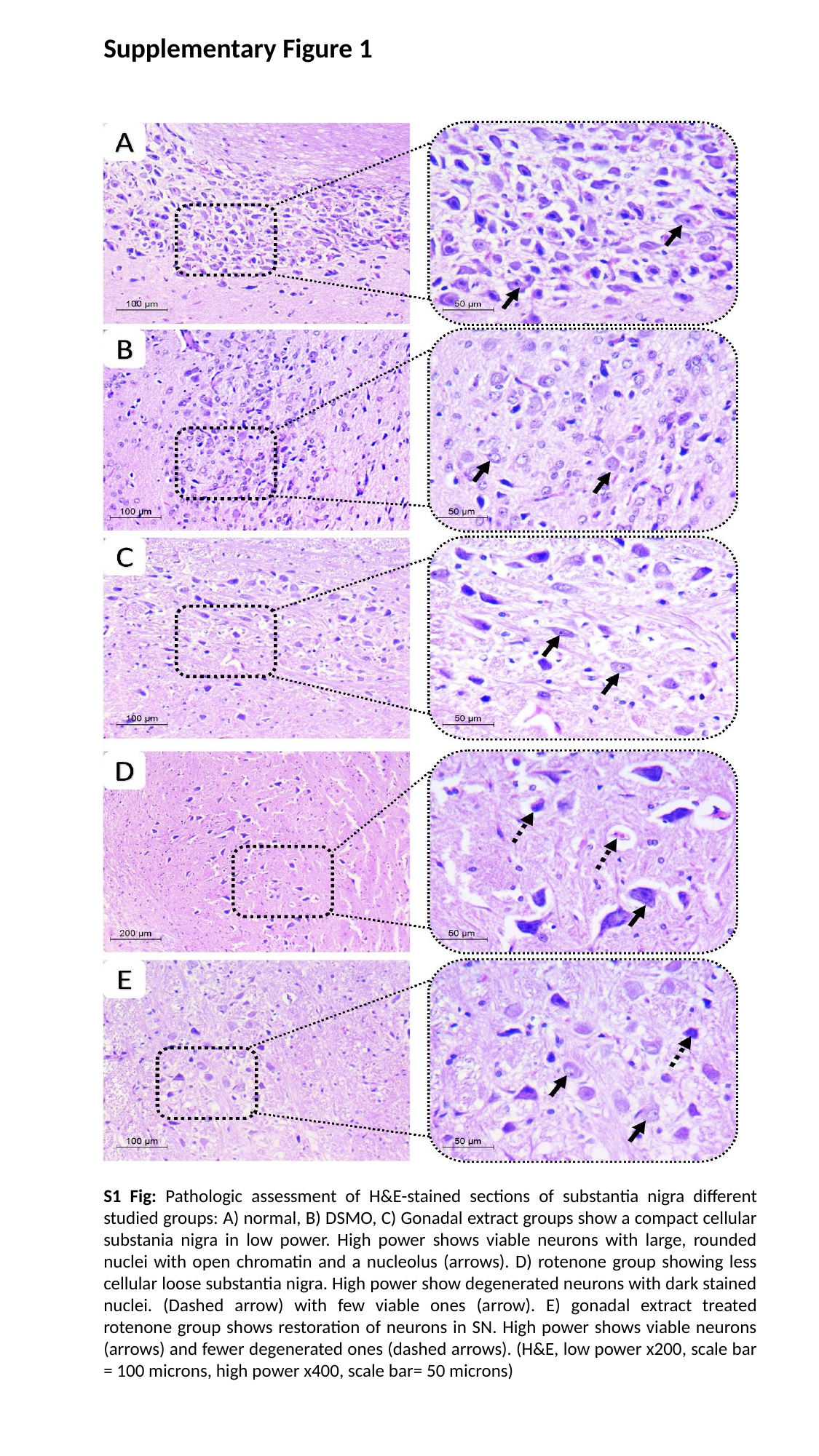

Supplementary Figure 1
S1 Fig: Pathologic assessment of H&E-stained sections of substantia nigra different studied groups: A) normal, B) DSMO, C) Gonadal extract groups show a compact cellular substania nigra in low power. High power shows viable neurons with large, rounded nuclei with open chromatin and a nucleolus (arrows). D) rotenone group showing less cellular loose substantia nigra. High power show degenerated neurons with dark stained nuclei. (Dashed arrow) with few viable ones (arrow). E) gonadal extract treated rotenone group shows restoration of neurons in SN. High power shows viable neurons (arrows) and fewer degenerated ones (dashed arrows). (H&E, low power x200, scale bar = 100 microns, high power x400, scale bar= 50 microns)
