# Supplementary material for: Paracentrotus lividus sea urchin gonadal extract mitigates neurotoxicity and inflammatory signaling in a rat model of Parkinson’s disease
Source: PLoS One. 2024 Dec 18;19(12):e0315858. doi: 10.1371/journal.pone.0315858 (PMC11654954; doi:10.1371/journal.pone.0315858)
Supplement: S2 Fig — large number of dopaminergic neurons are seen in deep brown background in A) normal, B) DSMO, C) Gonadal extract groups. D) rotenone group showing markedly diminished number E) gonadal extract treated rotenone group shows restoration of neurons in SN. (IHC, x200, scale bar 100 microns, inset x100). (PPTX) [file pone.0315858.s002.pptx]

## Slide 1
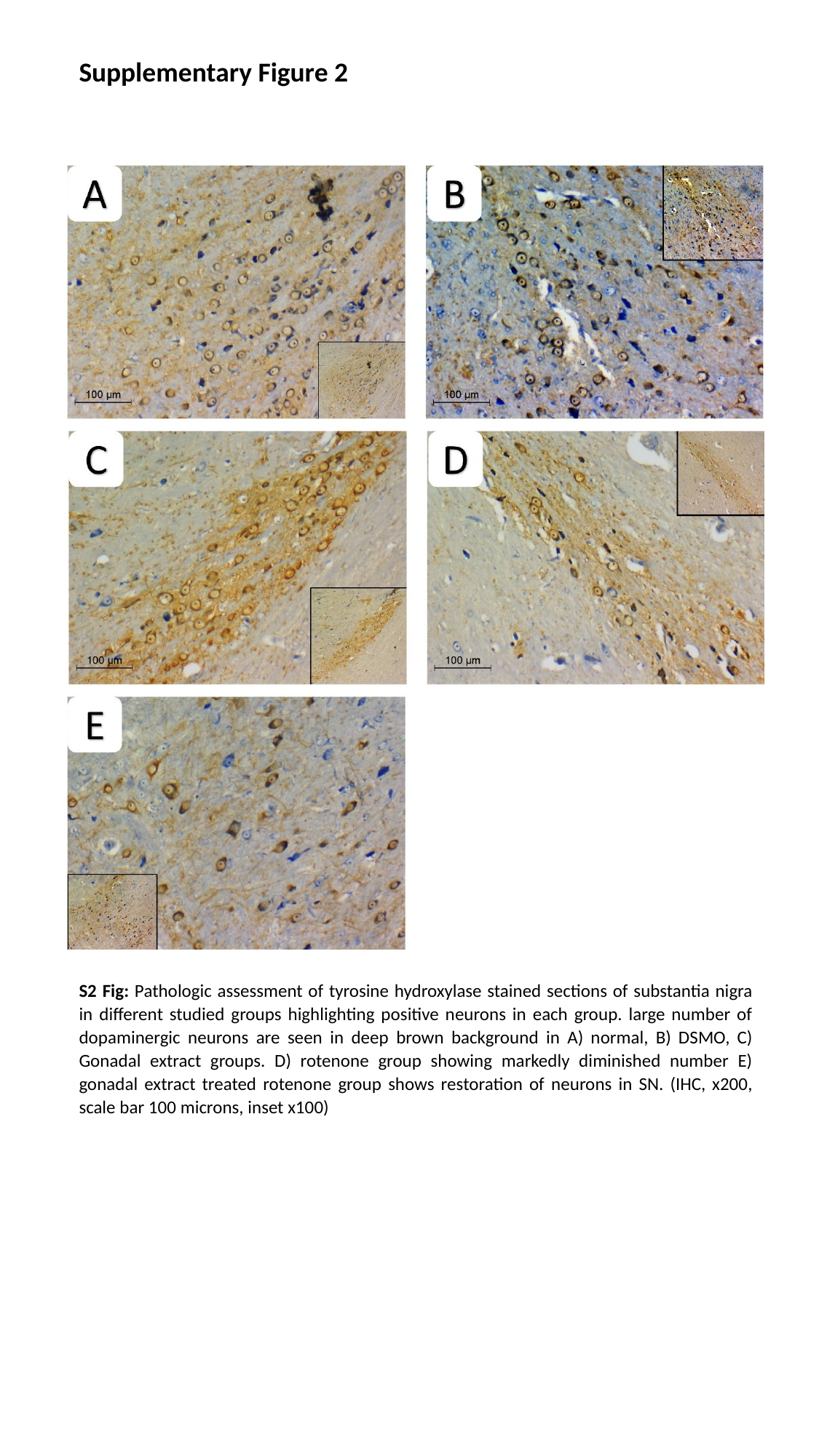

Supplementary Figure 2
S2 Fig: Pathologic assessment of tyrosine hydroxylase stained sections of substantia nigra in different studied groups highlighting positive neurons in each group. large number of dopaminergic neurons are seen in deep brown background in A) normal, B) DSMO, C) Gonadal extract groups. D) rotenone group showing markedly diminished number E) gonadal extract treated rotenone group shows restoration of neurons in SN. (IHC, x200, scale bar 100 microns, inset x100)
