# Supplementary material for: Paracentrotus lividus sea urchin gonadal extract mitigates neurotoxicity and inflammatory signaling in a rat model of Parkinson’s disease
Source: PLoS One. 2024 Dec 18;19(12):e0315858. doi: 10.1371/journal.pone.0315858 (PMC11654954; doi:10.1371/journal.pone.0315858)
Supplement: S3 Fig — negative staining of neurons is seen in A) normal, B) DSMO, C) Gonadal extract groups. D) rotenone group showing multiple positive alpha synuclein neurons (arrows) E) gonadal extract treated rotenone group decrease expression. (IHC, x400, scale bar 50 microns). (PPTX) [file pone.0315858.s003.pptx]

## Slide 1
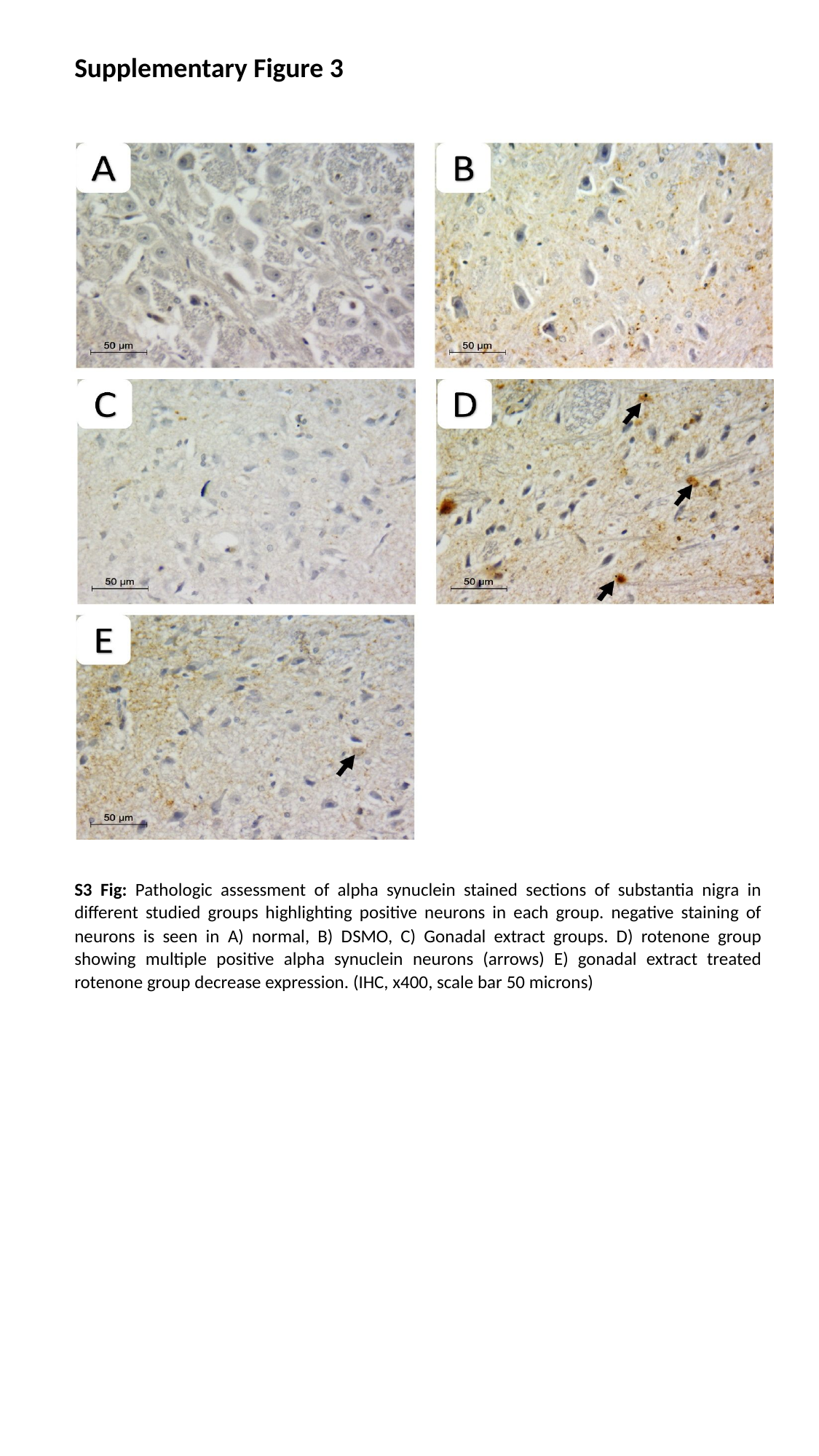

Supplementary Figure 3
S3 Fig: Pathologic assessment of alpha synuclein stained sections of substantia nigra in different studied groups highlighting positive neurons in each group. negative staining of neurons is seen in A) normal, B) DSMO, C) Gonadal extract groups. D) rotenone group showing multiple positive alpha synuclein neurons (arrows) E) gonadal extract treated rotenone group decrease expression. (IHC, x400, scale bar 50 microns)
