# Supplementary material for: Paracentrotus lividus sea urchin gonadal extract mitigates neurotoxicity and inflammatory signaling in a rat model of Parkinson’s disease
Source: PLoS One. 2024 Dec 18;19(12):e0315858. doi: 10.1371/journal.pone.0315858 (PMC11654954; doi:10.1371/journal.pone.0315858)
Supplement: S4 Fig — Pathologic assessment of H&E-stained sections of striatum different studied groups: A) normal, B) DSMO, C) Gonadal extract groups show a cellular striatum. High power shows viable neurons with large, rounded nuclei with open chromatin and a nucleolus (black arrows). few microglial cells (red arrows) and thin capillaries are seen (v) D) rotenone group showing evident disturbed architecture and hypocellularity. High power show few viable neurons (black arrows), multiple degenerated neurons with dark stained nuclei (Dashed arrow). The background shows vacuolated neuropil (star) and increased microglial cells (red arrows) E) gonadal extract treated rotenone group shows improvement of architecture of striatum. High power shows viable neurons (arrows) with residual focal neuropil vacuolation (star). Few microglial cells are seen (red arrows) (H&E, low power x200, scale bar = 100 microns, high power x400, scale bar = 50 microns). (PPTX) [file pone.0315858.s004.pptx]

## Slide 1
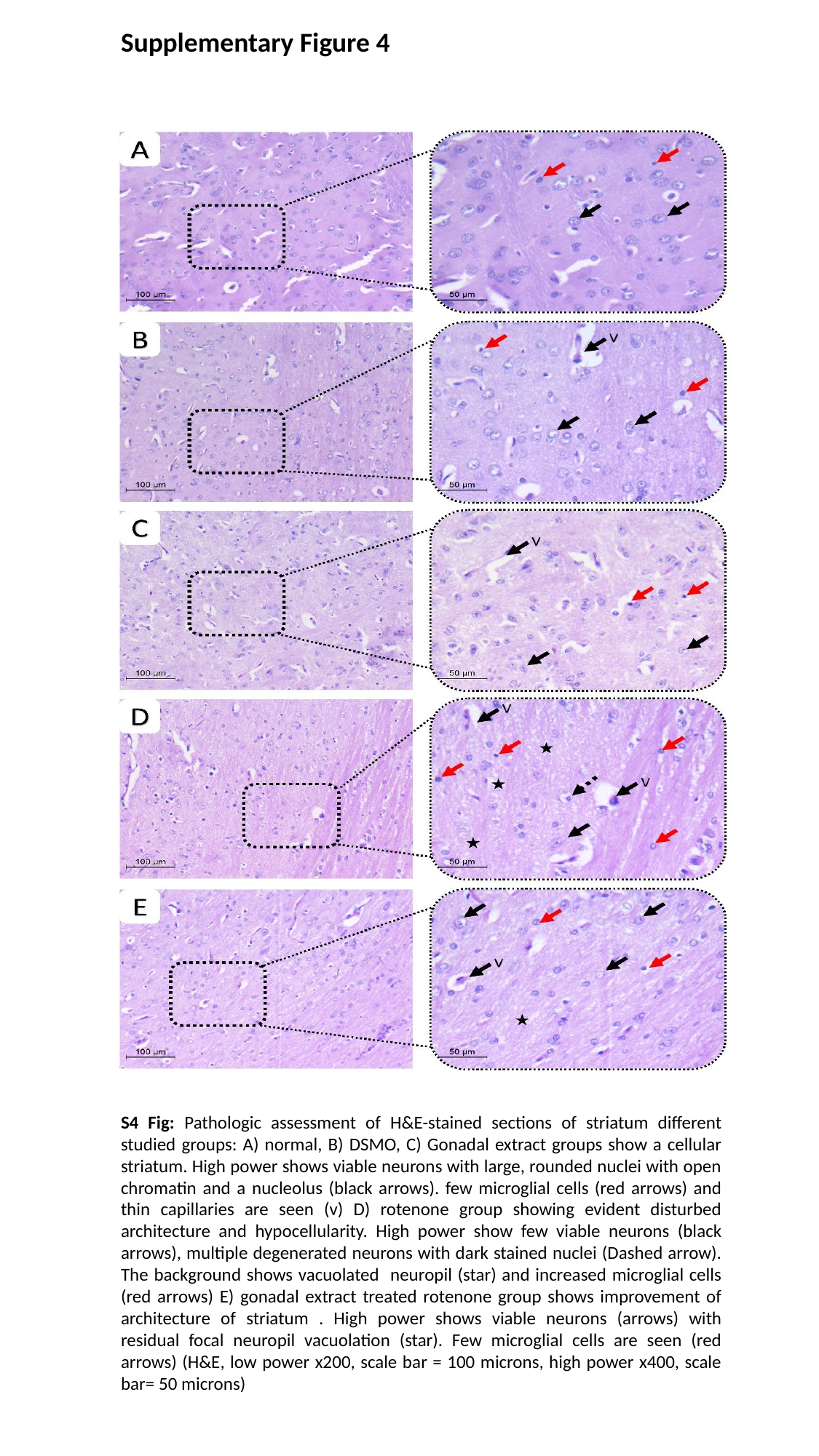

Supplementary Figure 4
S4 Fig: Pathologic assessment of H&E-stained sections of striatum different studied groups: A) normal, B) DSMO, C) Gonadal extract groups show a cellular striatum. High power shows viable neurons with large, rounded nuclei with open chromatin and a nucleolus (black arrows). few microglial cells (red arrows) and thin capillaries are seen (v) D) rotenone group showing evident disturbed architecture and hypocellularity. High power show few viable neurons (black arrows), multiple degenerated neurons with dark stained nuclei (Dashed arrow). The background shows vacuolated neuropil (star) and increased microglial cells (red arrows) E) gonadal extract treated rotenone group shows improvement of architecture of striatum . High power shows viable neurons (arrows) with residual focal neuropil vacuolation (star). Few microglial cells are seen (red arrows) (H&E, low power x200, scale bar = 100 microns, high power x400, scale bar= 50 microns)
