# Supplementary material for: Paracentrotus lividus sea urchin gonadal extract mitigates neurotoxicity and inflammatory signaling in a rat model of Parkinson’s disease
Source: PLoS One. 2024 Dec 18;19(12):e0315858. doi: 10.1371/journal.pone.0315858 (PMC11654954; doi:10.1371/journal.pone.0315858)
Supplement: S6 Fig — high density deep brown background is seen in A) normal, B) DSMO, C) Gonadal extract groups. D) rotenone group showing decreased staining density. E) gonadal extract treated rotenone group shows increased staining. (IHC, x200, scale bar 100 microns, inset x100). (PPTX) [file pone.0315858.s006.pptx]

## Slide 1
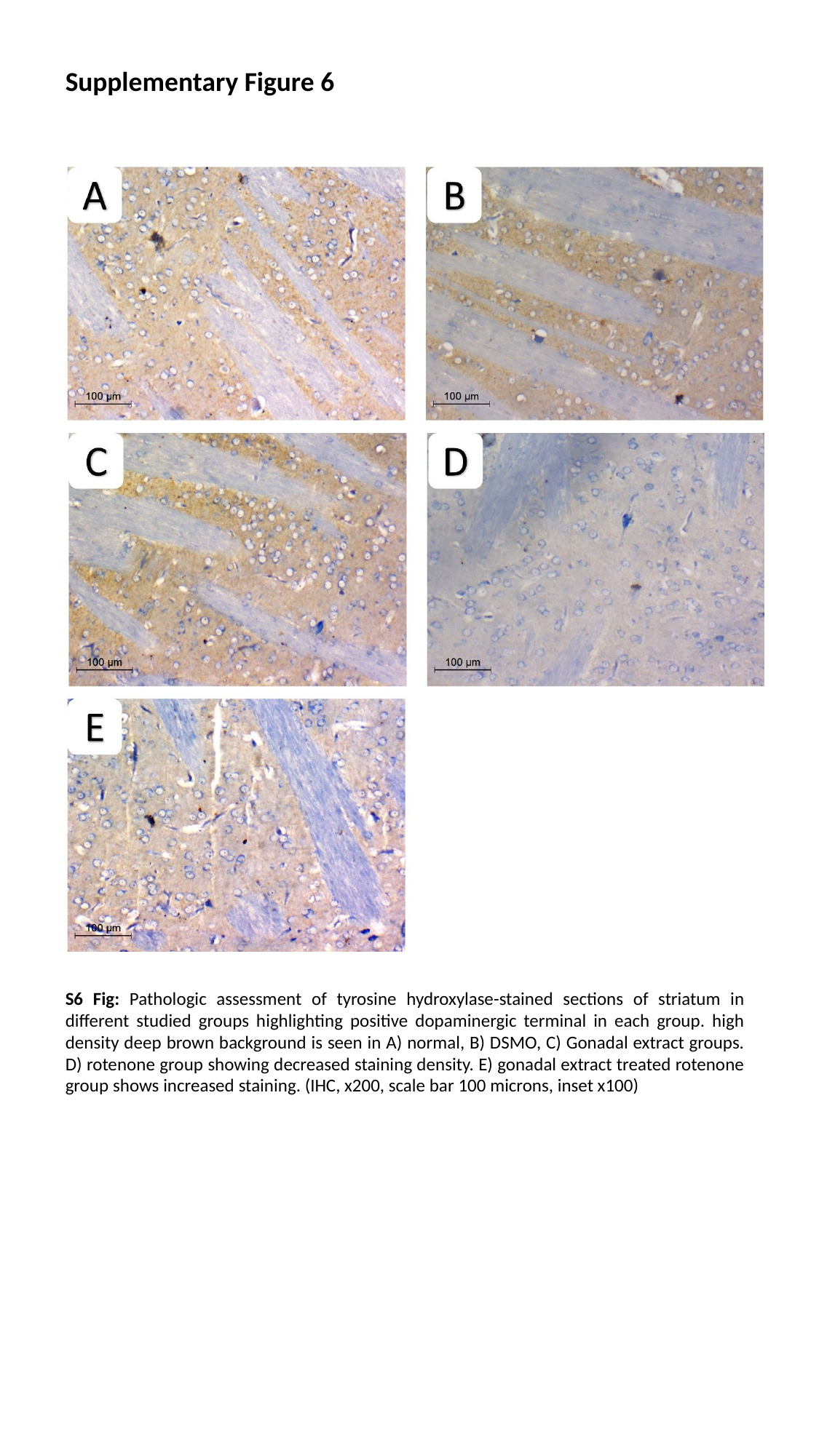

Supplementary Figure 6
S6 Fig: Pathologic assessment of tyrosine hydroxylase-stained sections of striatum in different studied groups highlighting positive dopaminergic terminal in each group. high density deep brown background is seen in A) normal, B) DSMO, C) Gonadal extract groups. D) rotenone group showing decreased staining density. E) gonadal extract treated rotenone group shows increased staining. (IHC, x200, scale bar 100 microns, inset x100)
