# Supplementary material for: Paracentrotus lividus sea urchin gonadal extract mitigates neurotoxicity and inflammatory signaling in a rat model of Parkinson’s disease
Source: PLoS One. 2024 Dec 18;19(12):e0315858. doi: 10.1371/journal.pone.0315858 (PMC11654954; doi:10.1371/journal.pone.0315858)
Supplement: S7 Fig — All the data were analyzed using one-way ANOVA followed by Tukey Pairwise Comparisons. Values are expressed as mean ± SE; n = 5 rats for each group. Different superscripts on the columns are significantly different at p≤0.05. (PPTX) [file pone.0315858.s007.pptx]

## Slide 1
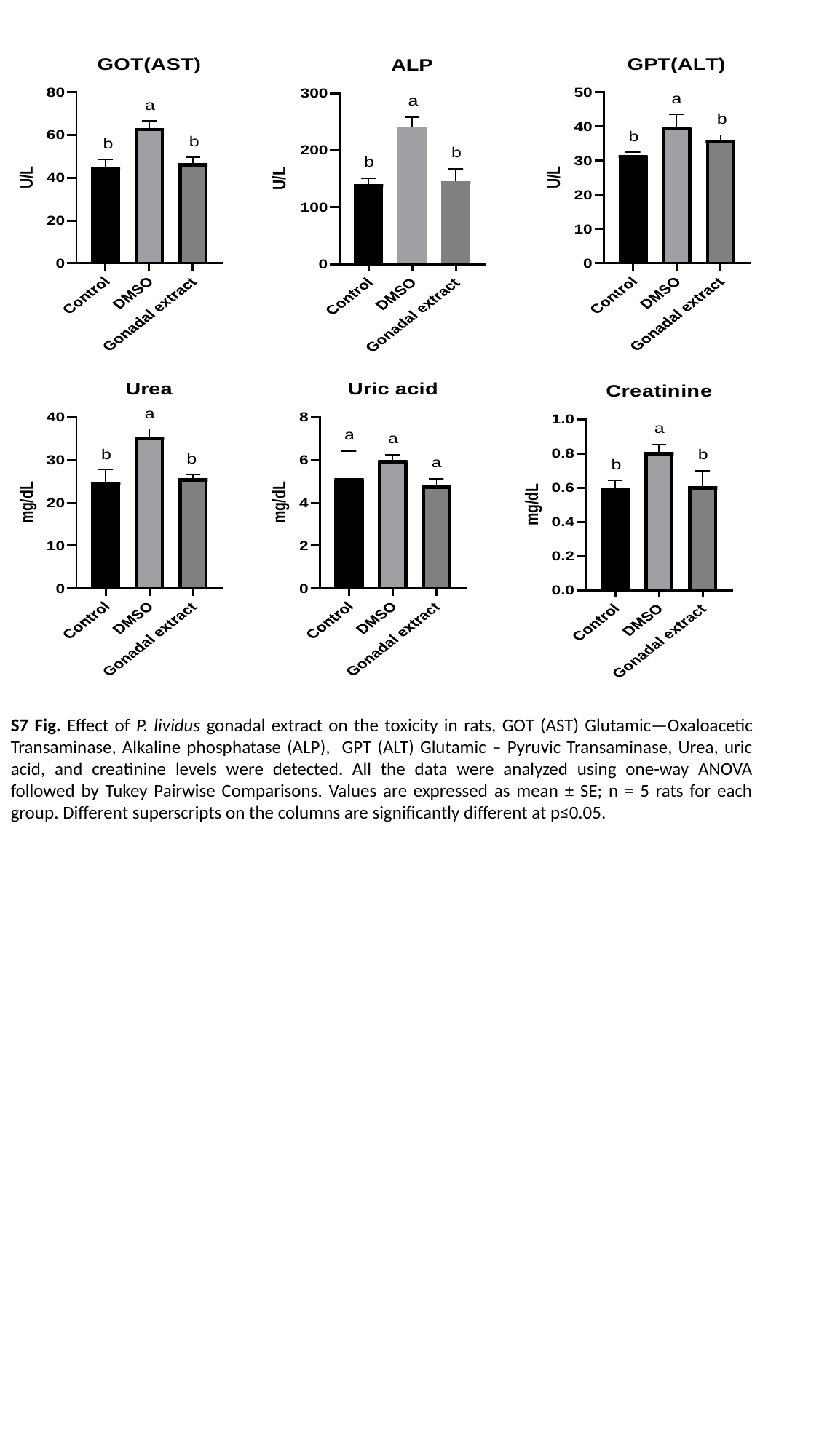

S7 Fig. Effect of P. lividus gonadal extract on the toxicity in rats, GOT (AST) Glutamic—Oxaloacetic Transaminase, Alkaline phosphatase (ALP), GPT (ALT) Glutamic – Pyruvic Transaminase, Urea, uric acid, and creatinine levels were detected. All the data were analyzed using one-way ANOVA followed by Tukey Pairwise Comparisons. Values are expressed as mean ± SE; n = 5 rats for each group. Different superscripts on the columns are significantly different at p≤0.05.
