# Supplementary material for: Paracentrotus lividus sea urchin gonadal extract mitigates neurotoxicity and inflammatory signaling in a rat model of Parkinson’s disease
Source: PLoS One. 2024 Dec 18;19(12):e0315858. doi: 10.1371/journal.pone.0315858 (PMC11654954; doi:10.1371/journal.pone.0315858)
Supplement: S8 Fig — A, B control, C, D DMSO, E, F P. lividus gonadal extract. No pathologic changes were detected in both organs in all studied rats. (PPTX) [file pone.0315858.s008.pptx]

## Slide 1
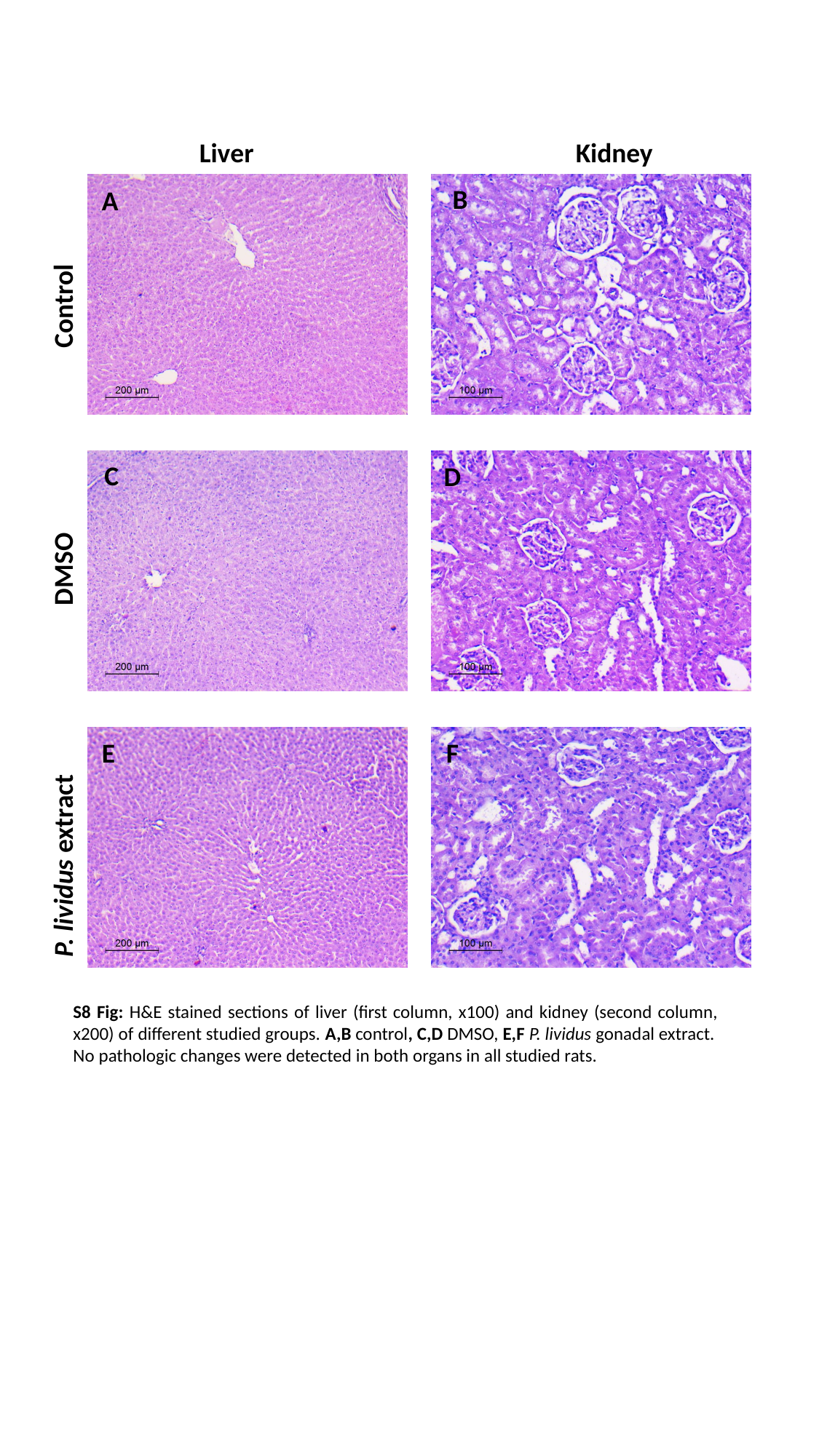

Liver Kidney
B
A
Control
C
D
DMSO
E
F
P. lividus extract
S8 Fig: H&E stained sections of liver (first column, x100) and kidney (second column, x200) of different studied groups. A,B control, C,D DMSO, E,F P. lividus gonadal extract. No pathologic changes were detected in both organs in all studied rats.
