# Supplementary material for: Paracentrotus lividus sea urchin gonadal extract mitigates neurotoxicity and inflammatory signaling in a rat model of Parkinson’s disease
Source: PLoS One. 2024 Dec 18;19(12):e0315858. doi: 10.1371/journal.pone.0315858 (PMC11654954; doi:10.1371/journal.pone.0315858)
Supplement: S1 Table — (DOCX) [file pone.0315858.s009.docx]

**Toxicity study:**

**Methods**

**Liver and kidney functions detection.**

To test the long-term toxicity of *P. lividus* gonadal extract in the treated rats, GOT (AST) Glutamic—Oxaloacetic Transaminase, Alkaline phosphatase (ALP), and GPT (ALT) Glutamic – Pyruvic Transaminase levels were detected, indicating liver function. In contrast, Urea, uric acid, and creatinine levels were detected, indicating kidney function.

Blood samples were collected from treated rats, serum was isolated and used in the detection.

Supplementary Table 1. List of liver and kidney function tests used to ass toxicity of *P. lividus* gonad extract

|  | Used kits | References |
| --- | --- | --- |
| GOT (AST) Glutamic – Oxaloacetic Transaminase | GOT (AST)  Glutamic – Oxaloacetic Transaminase. Colorimetric Method.  **Biodiagnostic** CAT. NO. AS 10 61 (45). | (1) |
| Alkaline phosphatase (ALP) | ALKALINE PHOSPHATASE  Colorimetric Method.  **Biodiagnostic** CAT. NO. AP 10 20 | (2) |
| GPT (ALT) Glutamic – Pyruvic Transaminase | GPT (ALT)  Glutamic – Pyruvic Transaminase.  Colorimetric Method.  **Biodiagnostic** CAT. NO. AL 10 31 (45) | (1) |
| Urea | UREA *Urease-Berthelot Method*  Enzymatic Colorimetric Method  **Biodiagnostic** CAT. NO. UR 21 10 | (3) |
| Uric acid | URIC ACID  Enzymatic Colorimetric Method  **Biodiagnostic** CAT. NO. UA 21 20 | (4) |
| Creatinine | CREATININE  Colorimetric Method. (End Point)  **Biodiagnostic** CAT. NO. CR 12 50 | (5) |

**Histological study**

Liver and renal tissues were harvested after sacrifice and fixed in 10% formalin. They were processed into paraffin blocks. Five microns thick sections were cut and mounted on glass slides to be stained by H&E stain. Sections were examined under light microscopy for any pathologic changes.

**Results**:

**Liver and kidney function detection.**

It was found that treatment with *P. lividus* gonadal extract didn’t increase liver and kidney function parameters compared to the control group. The obtained data were statistically analyzed using a one-way ANOVA test followed by Tukey Pairwise Comparisons (Figure 1 supplementary data).

**Histological study**

liver tissues of all studied groups showed preserved architecture. no inflammation or degenerative changes were detected. Renal tissues showed histologically free glomeruli. The interstitium showed closely packed tubules with no inflammation or fibrosis.

**References:**

1. Reitman S, Frankel S. A Colorimetric Method for the Determination of Serum Glutamic Oxalacetic and Glutamic Pyruvic Transaminases. American Journal of Clinical Pathology. 1957;28(1):56-63.

2. Belfield A, Goldberg D. Revised assay for serum phenyl phosphatase activity using 4-amino-antipyrine. Enzyme. 1971;12(5):561-73.

3. Fawcett JK, Scott JE. A RAPID AND PRECISE METHOD FOR THE DETERMINATION OF UREA. Journal of Clinical Pathology. 1960;13(2):156-9.

4. Barham D, Trinder P. An improved colour reagent for the determination of blood glucose by the oxidase system. Analyst. 1972;97(1151):142-5.

5. Schirmeister J. Determination of creatinine in serum. Dtsch Med Wschr. 1964;89(1940):796.
